# Evaluating energy consumption patterns in novel foamed ternary alkali-activated masonry blocks

**DOI:** 10.1038/s41598-024-80434-x

**Published:** 2025-01-16

**Authors:** Sunil Nandipati, Nikhil Kumar Degloorkar, Gandhi Pullagura, Debabrata Barik, Prabhu Paramasivam, Essam Althaqafi, Saiful Islam, Osamah J. Al-sareji

**Affiliations:** 1 Department of Civil Engineering, GITAM School of Technology, Visakhapatnam, 530045 India; 2 Department of Civil Engineering, Anurag University, Hyderabad, Telangana India; 3 Department of Mechanical Engineering, GITAM School of Technology, Visakhapatnam, 530045 India; 4 Department of Mechanical Engineering, Karpagam Academy of Higher Education, Coimbatore, 641021 India; 5 Centre for Energy and Environment, Karpagam Academy of Higher Education, Coimbatore, 641021 India; 6 Department of Research and Innovation, Saveetha School of Engineering, SIMATS, Chennai, Tamilnadu 602105 India; 7 Civil Engineering Department, College of Engineering, King Khalid University, 61421 Abha, Saudi Arabia; 8 Sustainability Solutions Research Lab, Faculty of Engineering, University of Pannonia, Egyetem Str. 10, Veszprém H, 8200 Veszprém, Hungary

**Keywords:** Foamed ternary alkali activated blocks, Energy efficiency, eQuest, Ceramic tile dust, Sustainable building material, Civil engineering, Mechanical engineering

## Abstract

This study endeavors to tackle the energy requirements of the building sector by employing passive design strategies. However, there exists a dearth of comprehension regarding the energy efficiency performance of foamed alkali-activated materials. To bridge this research gap, the study proposes a solution in the form of a thermally proficient wall material crafted from ceramic tile dust (CTD), class C fly ash (FA), and Ground Granulated Blast-Furnace Slag (GGBS), all of which are industrial by-products. The foamed ternary alkali-activated (FTAA) blocks, developed as a result of this research, exhibited commendable performance in terms of mechanical strength of 18.6 MPa, lower density of 1200 kg/m^3^, porosity of 15.95%, lower specific heat capacity (SHC) of 831 J/(Kg·K), and thermal conductivity (TC) of 0.38 W/(m·K). The thermal efficiency of FTAA blocks curtails the transfer of heat from the external environment to the interior, thereby engendering a more agreeable indoor milieu for occupants. A simulation study utilizing the eQuest tool was executed to evaluate the thermal attributes of the developed blocks and their consequential impact on energy requirements. The findings revealed that in comparison to clay bricks, employing FTAA blocks could yield potential annual energy savings of approximately 4%. Furthermore, notable cost savings of about 4.94% during peak summer months and 5.51% annually were observed. The significance of utilizing these ternary blocks, derived from industrial waste, resides in their affirmative contribution to environmental preservation, augmented indoor thermal comfort, and diminished energy consumption for end users. Consequently, this research makes a meaningful stride towards diminishing operational energy in buildings, harmonizing with sustainability objectives.

## Introduction

In recent years, the surge in construction activities in both urban and rural areas has led to a significant increase in energy demand. The energy consumed throughout the various phases of construction is substantial, encompassing pre-construction, construction, and post-construction stages. This surge in energy consumption can be attributed to factors such as climate change, economic growth, changes in building materials, and technological advancements^1^. Consequently, the sustainability of the construction building sector is being compromised. It is estimated that the building and construction sectors contribute over 30% to global energy consumption, with a substantial portion (40–60%) being allocated to heating, ventilation, and cooling (HVAC) systems^2,3^. Notably, the peak energy demand occurs during the summer months in regions of India with warm and humid climates, where temperatures often exceed 35 °C. In such conditions, energy is primarily required for space cooling, involving the usage of air conditioning and ventilation fans^4^. Unfortunately, the majority of India’s energy requirements are met through non-renewable sources like coal and nuclear energy, which contribute to greenhouse gas emissions and harm the ecosystem. Addressing this issue necessitates the collective effort of building stakeholders and governments to prioritize sustainable approaches and recommend strategies that reduce the energy demands of buildings.

The necessity for effective cooling in buildings in India is one of the main energy demands to enhance the thermal comfort of the inhabitants. Reduced heat transmission through the building envelope’s components should be achieved by using passive design techniques throughout the project’s architectural and construction stages. Thermal insulators such as XPS, EPS, polyurethane foam, fiberglass, and aerogel blankets are frequently employed during building to reduce the amount of energy needed for structures to meet their operational needs^5,6^. Several^7–9^ have reported research work on employing thermal insulation layers for the reduction of energy requirements and enhancing thermal comfort for the occupants.

Similarly, Lian et al.^7^ established rigid polyurethane foam for enhancing the both thermal as well as mechanical properties of a building’s envelope. Rojas et al.^8^ reported in the domain of thermal insulation for the use in building blocks employing the residual fibers from corn husk and wheat straw. This approach helped in the reduction of thermal conductivity in comparison with the expanded polystyrene insulation materials. It led to improvements in energy efficiency as well as thermal comfort in homes. Mujeebu et al.^9^ reported 14% annually in energy savings through the usage of nano aerogel glazing when comparing its energy efficiency to double-glazing windows. Using used coffee grounds as an insulating ingredient in plaster composites, Lachheb et al.^10^ were able to save heating and cooling requirements by 20% over traditional materials. Reducing density and heat conductivity, Bakatovich & Gaspar^11^ looked into thermal insulation panels using sphagnum moss and straw fibers.

Though it takes specific knowledge and additional resources, the use of thermal insulation materials has not received much attention in India despite earlier research efforts. By integrating industrial waste materials into building construction and systems, significant reductions in both embodied and operational energy can be achieved. These sustainable approaches not only contribute to reducing waste and lowering greenhouse gas emissions but also enhance the overall energy efficiency of the building. When designed thoughtfully, buildings that utilize industrial waste can be both environmentally friendly and cost-effective. One advised method of attaining sustainability is to use supplemental cementitious materials (SCMs) as substitute binders^12^. Waste materials from Industry and farming such as slag, fly ash (FA), ceramic waste, rice husk ash (RHA), and bottom ash (BA) can be utilized as SCMs^13–15^.

Alternatives to cementitious materials high in energy are always being sought by researchers. First proposed by Glukhovsky,^16^, alkali-activated materials have been offered as a potential long-term answer in this search. These compounds are formed by the reaction of binder and alkalis (like NaOH or KOH). Strong in calcium, alkali-activated binders also include significant amounts of silica and alumina. A gel with a tobermorite crystalline structure is produced when alkali-activated compounds react with calcium-rich binders. In the gel synthesis of this tobermorite structure, Ca^2+^, Si, H_2_O, and Na^+^ are included; Al^3+^ replaces and balances the negatively charged alkaline cations at tetrahedral units^17^. Moreover, a secondary gel known as N-A-S–H gel may be formed by adding alkali metals like Na. The alkali-activated matrix in these materials is strengthened in part by the tetrahedral polymeric chain of alumina-silicate.

Many scientists have investigated the mechanical, thermal, and physical properties of materials activated by alkali. To be employed as lightweight building materials, their density has been tried to be reduced^18,19^. By limiting heat transfer, the physical characteristics of these materials—such as density and porosity—as well as their thermal characteristics are crucial in preserving a cooling environment within buildings. To achieve low energy demands, it is essential to stress heat transfer reduction and appropriate insulation when selecting and developing composite materials for building projects^20,21^.

Different mixtures of foamed alkali-activated materials function effectively as building insulators. Both internal thermal loading brought on by heat transfer and outside thermal loading from heated walls present problems for many recently constructed buildings^22^. As such, it is essential to manage the external heat loading transfer with accuracy. Greater internal heat gain, higher energy consumption, and increased operational costs may all be consequences of inefficient designs^23^.

## Literature review

Development of the building envelope is a primary concern nowadays because sustainable or green rating initiatives concerning energy savings primarily focus on the thermal conductivity and specific heat of the whole building envelope^24,25^. Building façade needs to be studied based on the development of new types of bricks made from locally available waste material^26,27^. It is not given much attention. So, researchers have been taking steps forward, meticulously analyzing the incorporation of binary and ternary use of mixed wastes that can improve thermal performance without compromising the various functional performance parameters^28–30^. This literature review mainly provides insight into the development of walling systems by considering waste as a source material that can improve building performance in terms of energy efficiency^31,32^. Thus, this research provides comprehensive information and analyzes the previous literature on the influence of waste materials based on parameters such as compressive strength (CS), flexural strength (FS), water absorption (WA), density, etc. with thermal conductivity of bricks/blocks to develop more sustainable alternatively high-performance walling envelope. The overview of this study can serve as a basic reference for the construction fraternity working towards green initiatives and further improving overall performance in terms of thermal behavior by incorporating waste to develop an energy-efficient masonry brick. Detailed research needs to be carried out on the utilization of waste to enhance best practices on wall material based on the current production of bricks^33,34^.

Praburanganathan et al.^35–39^ carried out extensive research on using various categories of waste materials in making bricks/blocks. The research on bricks/blocks is very limited papers focusing on the overall performance of the use of agro-industrial and municipal solid wastes with a limited focus on thermal enhancement and life cycle energy. This comprehensive review mainly focused on 1. source category of waste (industrial, agro, and municipal solid waste, etc.) as suggested by Murmu & Patel^40^, 2. methods of manufacturing 3. physical and strength properties including specific gravity, water absorption, density, compressive and bending strength, etc. 4. Impact and material characterization of wastes used in the preparation^41,42^.

Researchers around the world are using various sources of waste materials especially, industrial, agricultural, municipal, and construction and demolition for manufacturing bricks^43,44^. Numerous researchers have examined the incorporation of municipal solid waste, agricultural waste, and industrial waste into bricks through the utilization of diverse production methods^45–47^. As part of sustainable practices, researchers should primarily concentrate on the life cycle energy of buildings by enhancing thermal comfort through an efficiently insulated wall system. While summarizing the research studies within the subsequent literature review, the emphasis is placed on the physical, mechanical, and thermal properties of bricks^48,49^. Classification is based on the two methods of manufacturing bricks i.e., 1. Fired bricks, and 2. Unfired bricks.

The compressive strength of conventional bricks, i.e., burnt clay bricks, is reported to be 3.5 MPa as per the standard IS 1077:1992^50^. Burnt clay bricks are made from excavated clay as the source material. Clay containing a high amount of silica and alumina will provide excellent bonding and strength after being fired at high temperatures between 700 and 1100 °C. According to Karaman et al.^51^, an increase in strength is mostly attributed to firing time, resulting in reduced water absorption, porosity, and increased density to some extent due to the elevated temperature. Therefore, the increase in strength of clay bricks depends not only on the extended firing time but also on observable changes in mineralogical properties and texture. A significant drawback of these bricks is their high energy consumption during manufacturing and substantial emissions of greenhouse gases, rendering their production unsustainable. From an environmental standpoint, numerous researchers have examined the advantages and disadvantages of reducing carbon emissions in fired bricks by incorporating various types of waste. Some researchers have taken a step further and conducted comprehensive investigations into the mechanical, physical, and thermal characteristics of burnt clay bricks.

Okunade et al.^52^ investigated the effects of sawdust and wood ash as admixtures by varying proportions to develop burnt laterite-clay bricks. They utilized percentage ratios of sawdust (0%, 2.5%, 5%, 7.5%, 10%) and wood ash (10%, 7.5%, 5%, 2.5%, 0%) to achieve a reduced density of 1512 kg/m^3^ from the original 1578 kg/m^3^. The wood ash and sawdust were combusted at an oven temperature of 600 °C. The findings indicated that using 10% wood ash in bricks increased compressive strength and decreased water absorption to a certain extent. The study has concluded that an increase in the percentage of sawdust has led to the opposite effect in the final product. This was mainly attributed to pore formation and decreased compactness.

Production of sustainable (unfired) bricks does not require a process of firing. It mainly relies on cementing properties of raw materials such as waste^53^. A lot of emphasis has been placed by researchers on the incorporation of the utilization of industrial and agricultural wastes to produce sustainable newly developed bricks with all qualitative standards as per requirements of conventional brick materials^54,55^. Generally, the preparation of unfired bricks is based on the use of conventional cement as the primary raw material in the production of cement bricks. In the cement bricks, the heat of hydration takes place due to the addition of water, which is responsible for setting and hardening key bonds such as calcium-silicate-hydrate(C–S–H) and calcium-alumina-silicate hydrate (C–A–S–H) gels in order to contribute the strength and durability.

Cement produces carbon emissions during the manufacturing process and high embodied energy. Incorporating wastes in cementitious bricks can reduce the release of greenhouse gases as well as the embodied energy of brick to a certain extent^56^. The physical and mechanical properties of fly ash bricks using corncob ash are measured with an optimum replacement of 10% of cement^57^. Thermal conductivity value decreases with an increase in corncob ash up to 30%.

The production of bricks through the process of firing has certain drawbacks due to the burning of wood at high temperatures leads to environmental problems^28^. Based on the drawbacks of firing clay bricks, new types of innovative and environmentally friendly bricks are developed through the process of geopolymerization. Geopolymerization takes place by utilization of industrial byproducts (FA, GGBS) as primary source material, which is rich in silica and alumina with a required quantity of alkaline solution at various curing conditions (ambient or steam or oven). Geopolymer has several additional advantages in terms of (strength, durability, thermal insulation, and low embodied energy) compared to firing and cementing. These insights on features have significantly made geopolymer a unique material in terms of enhancing sustainability.

The influence of GGBS and manufacture sand(M-sand) to manufacture geopolymer bricks with equivalent performance as conventional first-class A bric^58^ with various combinations of mix ratio, Sodium Silicate to Sodium Hydroxide and molarity. The results predict that the maximum compressive strength is 23.8 MPa for the 70% GGBS and 30% M-Sand under ambient curing conditions. GGBS mixed with an alkaline solution has produced quick setting and hardening properties due to the rich calcium oxide content in GGBS. Chithambar Ganesh et al., 2020,^58^ make use of industrial waste and M-sand has resolved issues related to waste disposal problems and also the scarcity of conventional river sand to produce energy-efficient eco-friendly bricks.

Morsy et al.^59^ recycled rice straw ash (RSA) influences the thermal conductivity and water absorption of geopolymer adobe bricks. The RSA was used in various replacements (0–20%) and SH contents (2.5–10%) after curing the bricks for 28 days. They concluded that increasing the percentage of RSA and SH increases the CS and decreases thermal performance, CS, and WA. Finally, it has been stated that optimum CS and thermal conductivity are 2.1 MPa and 0.46 W/m·K at 20% RSA and 10% SH. Thus, it is recommended that using this type of abode brick serves the purpose of minimizing the energy consumption needs of the building.

## Research objectives and methodology

The present work focuses primarily on the following objectives:

- Development of ternary alkali-activated (TAA) and foamed ternary alkali-activated (FTAA) blocks using ceramic tile dust waste, fly ash, GGBS, and protein-based foaming agent (for FTAA blocks).
- Physical, mechanical, and thermal properties of TAA and FTAA blocks are assessed and compared with conventional blocks namely clay and fly ash blocks.
- These TAA and FTAA blocks produced are compared with conventional walling materials in terms of energy efficiency.

Hence, from the above objectives, it is clear that the study mainly focuses on the usage of industrial waste (as a supplementary cementitious material) to develop alkali-activated masonry walling material to enhance the indoor thermal comfort levels of the occupants. The research findings and outcome provide a solution for better thermal comfort levels of occupants as part of a sustainable approach. The developed material ensures thermal insulation features over commercially available products in the market.

## Methodology

### Development of ternary alkali-activated (TAA) blocks

**Fig. 1:**
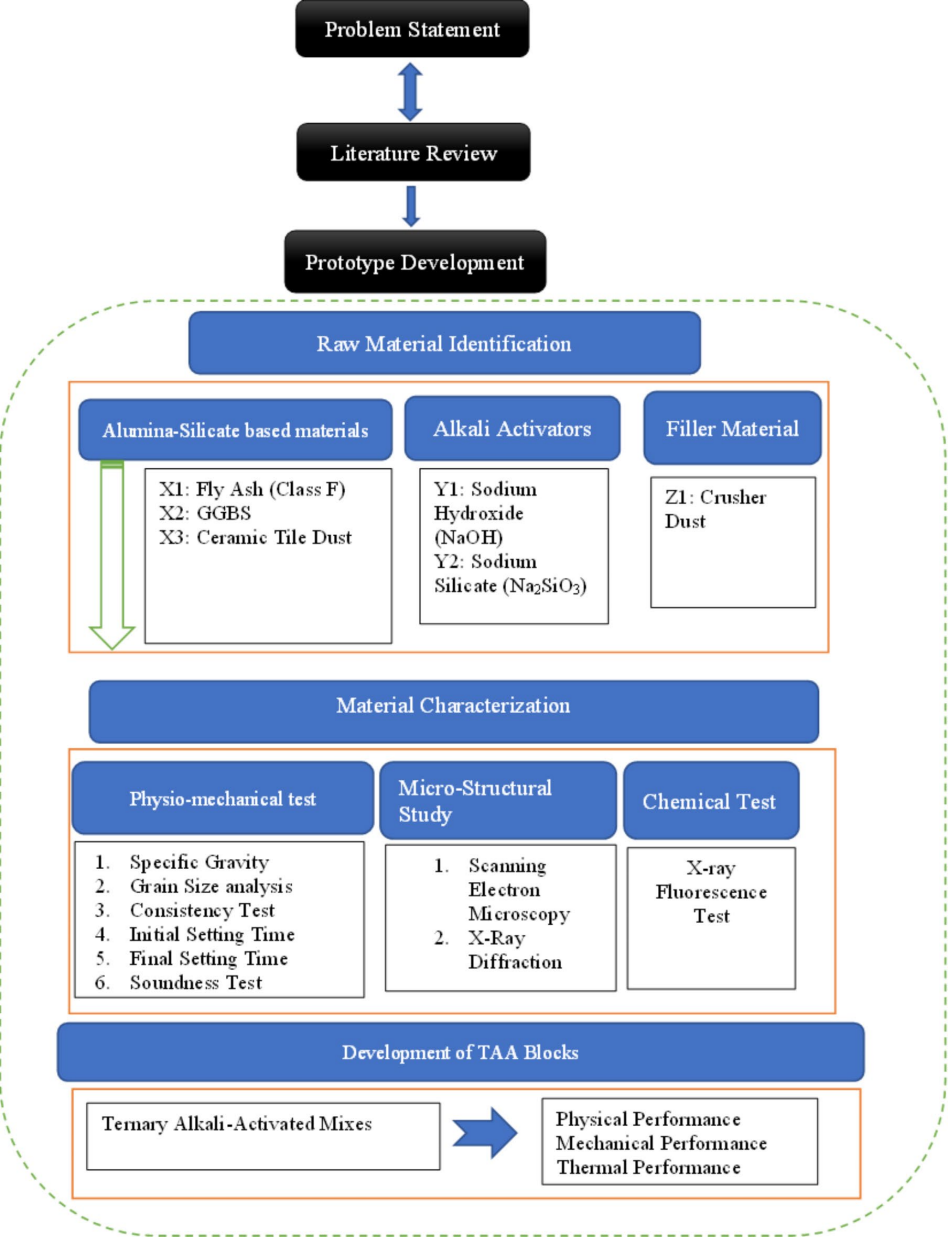
Flow chart of the development of TAA Blocks.

The product is made utilizing alumina-silicate raw material ceramic tile dust (CTD), a waste product of the ceramic industry. To help with the alkali activation process were also ground granulated blast furnace slag (GGBS) and fly ash (Class F). Two main components are used to generate the ternary alkali-activated (TAA) product: liquids and solids. The materials classified as solids include fly ash (Class F), crusher dust (CD), ground granulated blast furnace slag (GGBS), and ceramic tile dust (CTD). Alkaline activators include sodium hydroxide, sodium silicate, and water make up the liquid component. The flow chart of the development of TAA blocks is shown in Fig. 1.

**Fig. 2:**
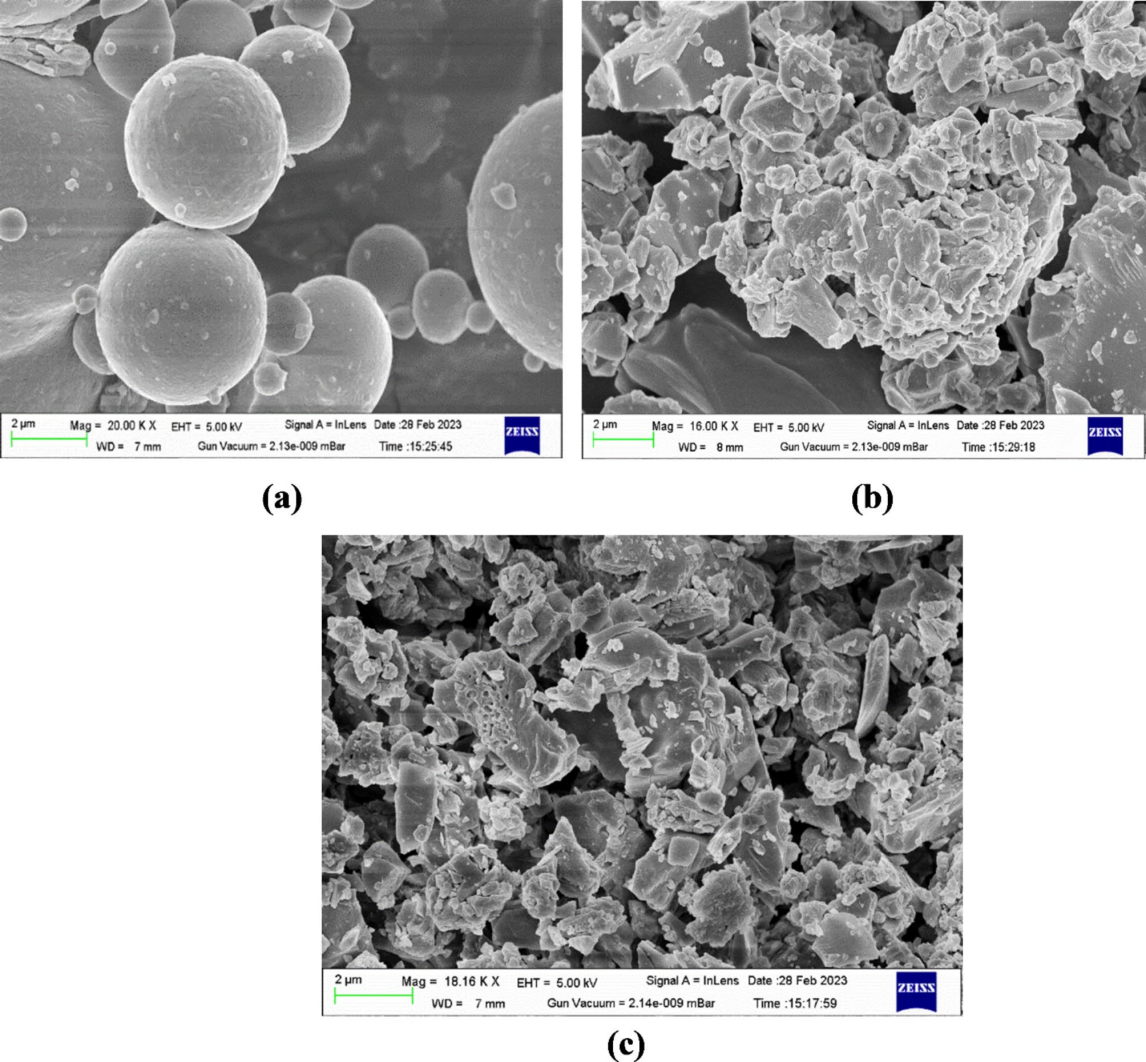
Images (SEM) of (**a**) FA (**b**) GGBS (**c**) CTD.

Table 1 lists the physical properties of FA, GGBS, and CTD. The corresponding test methods are referenced within the same table. The test results indicate that FA, GGBS, and CTD have the potential to be employed as alumina-silicate source materials. Chemical characterization of FA, GGBS, and CTD is conducted through X-ray Fluorescence (XRF) testing, and the results are provided in Table 2. The analysis reveals that these solid raw materials contain aluminum oxide (Al_2_O_3_), silica dioxide (SiO_2_), and calcium oxide (CaO), which play an important part during the synthesis of the alkali-activated matrix and contribute to the test material’s strength enhancement. Figure 2 depicts SEM images of FA, GGBS, and CTD, illustrating their respective morphologies. FA particles (Class F) exhibit a spherical shape with a smooth surface, while GGBS and CTD particles are characterized by flaky-elongated shapes, sharp-edged angularity, and irregular angular structures.

**Table 1:** Physical properties of Fly ash, GGBS, and Ceramic Tile Dust.

IS Method	FA	GGBS	CTD	
Grain size analysis				
Gravel (%)	IS 2720:198^60^	0	0	0
Sand (%)		12.7	24.8	15.1
Silt & Clay (%)		87.3	75.2	84.9
Consistency (%)	IS 1727:196^61^	29.8	25.5	33.7
Final setting time (Mins.)		425	321	262
Initial setting time (Mins.)		121	92	130
Specific gravity		2.15	2.85	2.51
Autoclave expansion (%)		NA	0.045	0.075

**Table 2:** Chemical Characterization.

Sample	FA (%)	GGBS (%)	CTD (%)
SiO_2_	61.34	34.25	59.62
TiO_2_	1.376	0.37	0.99
Al_2_O_3_	28.47	10.72	16.62
MnO	0.38	0.33	0.108
Fe_2_O_3_	4.10	0.55	3.52
CaO	1.11	34.88	5.72
MgO	0.40	10.98	4.533
Na_2_O	0.62	0.21	0.79
K_2_O	0.34	0.31	2.11
P_2_O_5_	0.622	0.171	0.123
SO_3_	0.26	7.139	3.806

Ceramic tile dust waste has an irregular and angular particle it has a tendency to create a larger surface area compared to spherical particles of a similar volume. The increased surface area enhances the availability of reactivity for the alkali-activation process. Due to active particles, it enhances participation in the polymerization process and allows slow reactions between the aluminosilicate components of the ceramic tile dust and the alkaline activators. By using GGBS, the polymerization process will be enhanced more quickly than CTD. To enhance better reactivity of blocks, it is better to prepare a ternary mix with FA, GGBS, and CTD.

To prepare the ternary alkali-activated (TAA) blocks, the alkali activators, Na_2_SiO_3_, and NaOH, are thoroughly mixed and stored in a closed, air-tight container for a day before casting the blocks. The liquid components, SH, and SS are blended with a Na_2_SiO_3_: NaOH ratio of 2.5, maintaining the NaOH molarity at 8 M. The solid ingredients are combined based on the mix proportion of FA: GGBS: CTD: CD, which is 0.15:0.25:0.1:0.5. For approximately 4–8 min, the solids and liquids must be uniformly mixed in a rotary mixer to achieve a homogeneous blend. Uniform mixing is essential for enhancing reactivity, and a longer duration of mixing improves workability and strength. After molding, the specimens are allowed to dry until the mix hardens.

Physical, mechanical, and thermal performance tests are conducted on two sizes of blocks: 100 × 100 × 100 mm^3^ and 100 × 100 × 500 mm^3^ (ASTM C20; ASTM C67-12)^62,63^. Additionally, cubes are subjected to outdoor curing for 28 days. Then tests are performed to evaluate the performance of the developed blocks in comparison to conventional clay bricks using the Hot Guarded Plate apparatus (ASTM C1044-16)^64^.

### Production of foamed ternary alkali-activated (FTAA) blocks

**Fig. 3:**
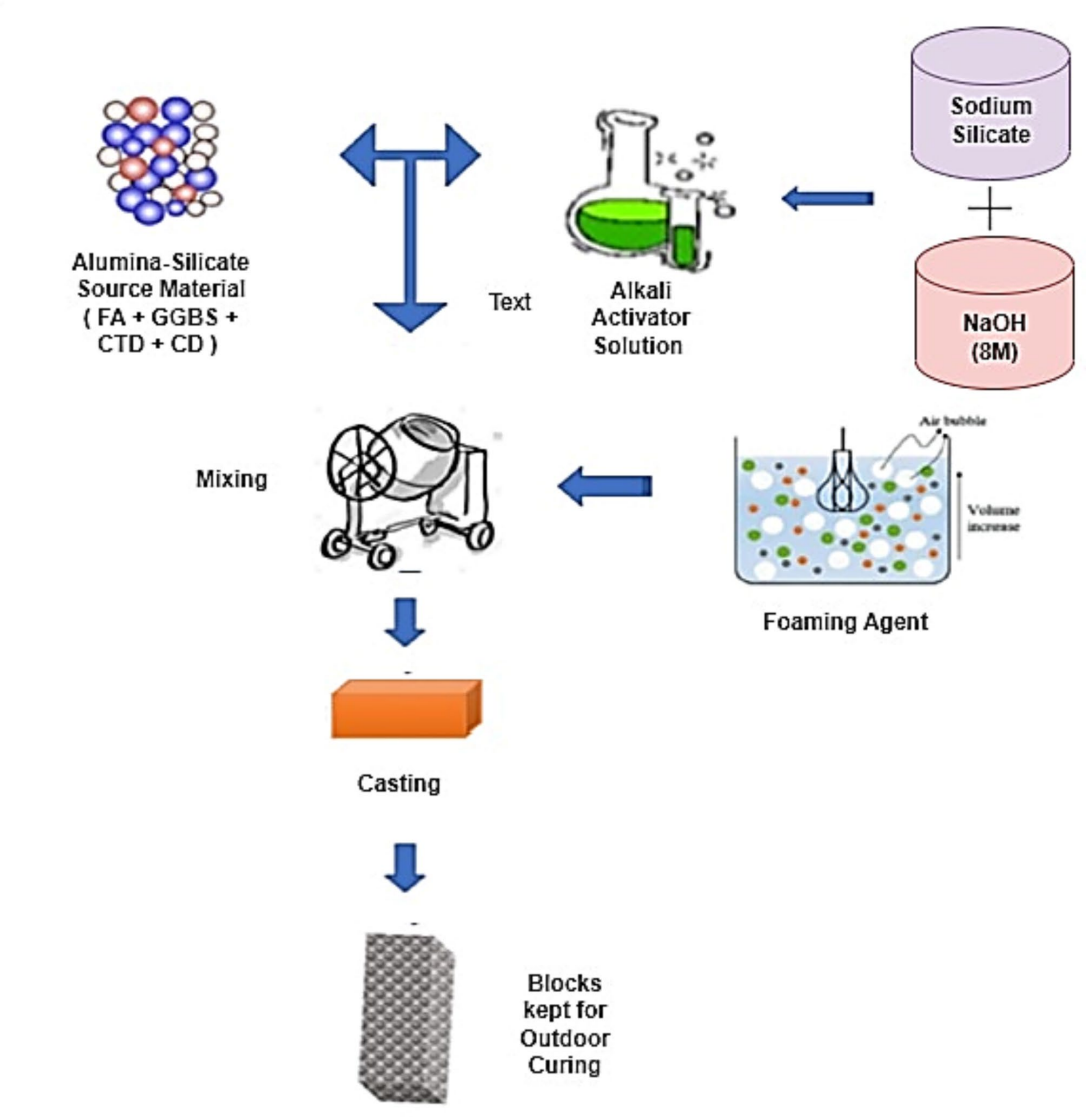
Schematics of FTAA blocks production.

**Fig. 4:**
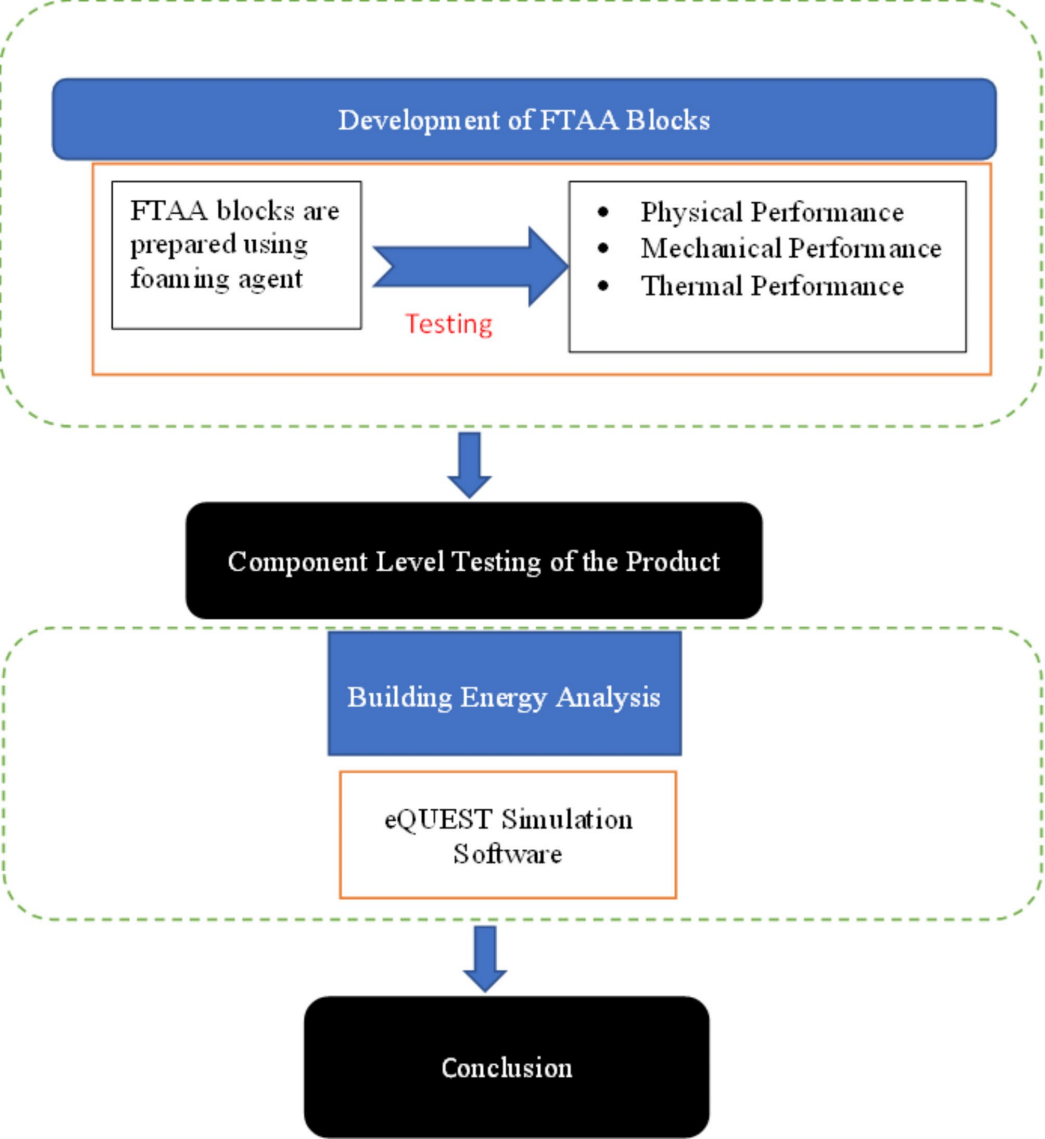
Flow chart of the development of FTAA blocks.

The preparation process of FTAA blocks closely follows the methodology used for TAA blocks. However, an additional protein-based foaming agent^65^ is incorporated in the development of FTAA blocks. During the block casting process, 3% of the total weight of the powder and 8% of water are introduced to the wet mix as an extra foaming agent, enhancing workability. The mixture is then moved to the mold, which generates air bubbles within the specimen, resulting in a porous nature with voids ranging in size from 1–2 mm. The molds containing the FTAA wet mix undergo a similar curing process as the TAA mix. All tests conducted for the TAA blocks are performed similarly for the FTAA blocks. Figure 3 depicts the flow diagram illustrating the preparation of FTAA blocks, it also visually presents the porous structure of the developed blocks. The flow chart for developing the FTAA blocks is indicated in Fig. 4.

**Fig. 5:**
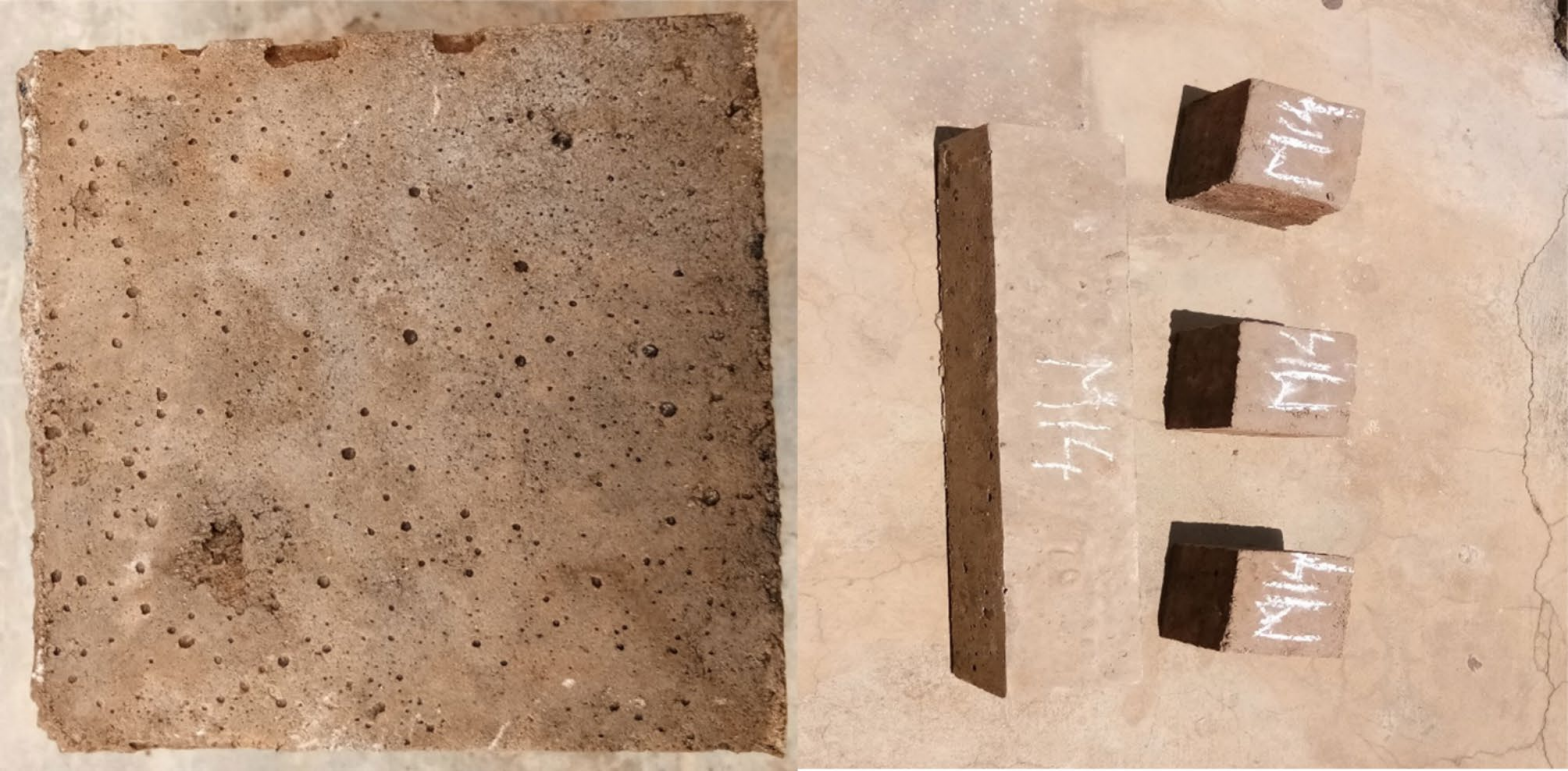
Images of the FTAA blocks.

The images of prisms and cubes cast for flexural and compressive strength of FTAA blocks is shown in Fig. 5.

### Thermal conductivity (TC) test

**Fig. 6:**
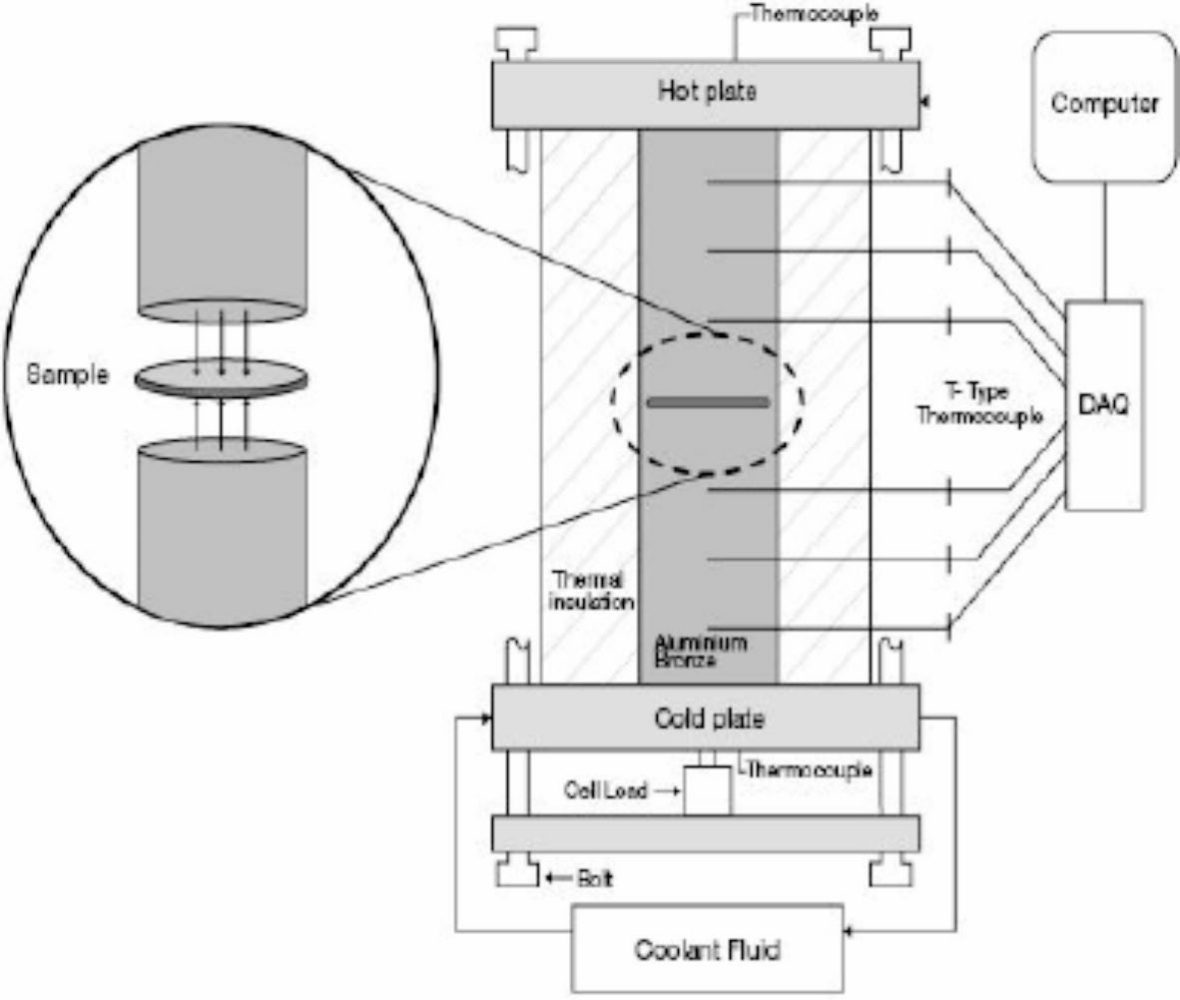
Schematic diagram of hot guarded plate apparatus (ASTM C1044, 2016)^64^.

**Fig. 7:**
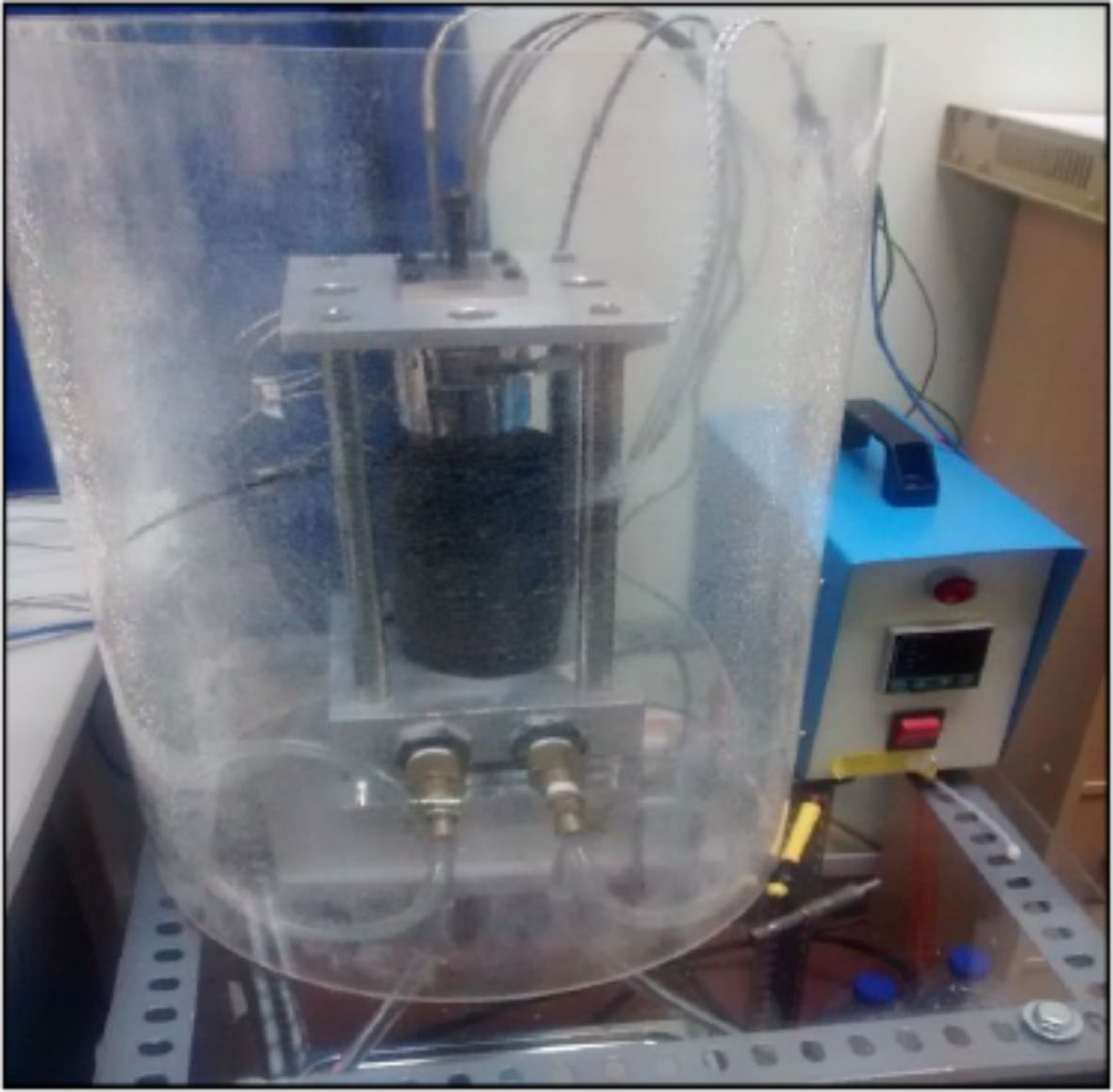
Hot Guarded plate apparatus.

The two different methods used for measuring thermal conductivity are transient and steady state. Conventionally, the former is commonly used for determining low thermal conductivity. However, in most cases, steady-state methods are not only accurate but also widely acceptable. For the determination of thermal conductivity, a hot guarded plate apparatus was used, the schematic diagram is shown in Fig. 6. For this test samples having circular disc shapes of 30 mm diameter and 5-7 mm thickness were used. The samples were cast and cured for 28 days for experimentation. A test rig having a working principle of hot guarded plate apparatus that was used is shown in Fig. 7. Samples were then transferred to the test rig for measurement of thermal conductivity. All the measurements were taken at room temperature.

Figure 7 shows a test rig that consists of two steel rods that were placed between each other. The upper rod was heated using an electric coil, while the lower one was cooled using a coolant fluid. Through the sample, the heat from the heated upper rod was transferred to the cold plate. Figure 7 shows the various locations where thermal sensors were placed to collect temperature data during the process of heat transfer. These readings were then recorded using a data acquisition system. The calculation of thermal conductivity was carried out using Fourier’s equation as indicated below as equation-1.

1 $$q= -k.A. \frac{dt}{dx}$$

where q is the steady-state flow, K is thermal conductivity, A is the cross-sectional area of the sample, and dt/dx is the temperature gradient.

Theoretical determination of thermal conductivity can be done by using the theoretical relation between dry density (Dd) and the thermal conductivity of the material^66^, as given in Eq. 2. Dry density can be used to predict the theoretical thermal conductivity of materials, as it can be used to compare and validate the experimental results.

2 $$k=0.0559 {e}^{(0.0014 Dd)}$$

### Specific heat capacity (SHC)

The specific heat capacity of a kilogram of material is determined by the amount of heat that it takes to raise its temperature by one kelvin. This is a vital factor that can be used in measuring thermal mass. Thermal analysis technique that probes into how the specific heat capacity (C_P_) of materials is altered by temperature is measured by a differential scanning calorimeter (DSC). When a known mass of a specimen is cooled or heated, the changes in its heat capacity are traced as changes in the heat flow. It is normally measured as the heat capacity at constant pressure (C_P_), but theoretical calculations are frequently reported in terms of the heat capacity at a constant volume (C_V_). The formula for the calculation of theoretical specific heat capacity is given in Eq. 3.

3 $$Q = C_{p} \times m \times \Delta T$$

where, Q = Heat added (J). Cp = Specific heat capacity (J/g·ºc). m = mass of material (g·), △T = Change in temperature (ºc).

The DSC measures a certain heat capacity by comparing the difference between a reference and a sample. A sample is placed on a carrier inside a heated furnace, which generates radial heat. Temperature is then detected by thermocouples.

### Energy performance evaluation using eQuest simulation model

To measure the energy performance of the building, an eQuest simulation model was created, enabling the study of energy consumption and total energy requirements (TER). The methodology involved several steps, including the construction of a model building by inputting the parameters (material properties of the envelope, lighting loads, and equipment loads) with the inclusion of weather data with the help of the utilization of an energy efficiency measuring wizard. By employing energy simulation studies based on DOE-2.2, graphical representations were generated, facilitating a comprehensive understanding of the building’s energy needs and overall efficiency. According the previous studies^67–69^, It is noticed that HVAC (Heating, Ventilation, and Air Conditioning) load is a critical factor for energy consumption per annual year. HVAC load depends on envelope design, HVAC efficiency, and thermostat setpoint.

**Fig. 8:**
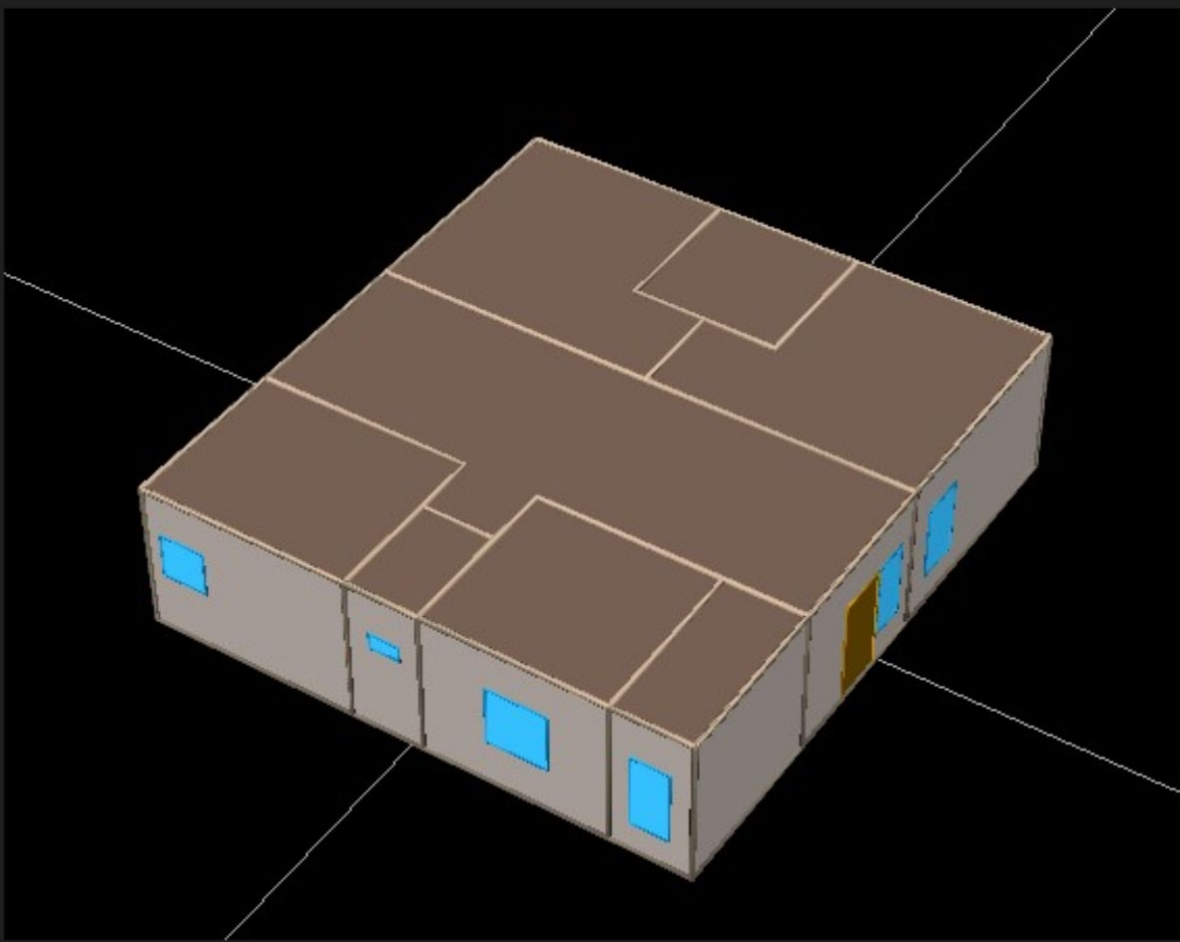
Energy simulation studies building model in eQuest software.

After creating the 3D model with a single floor area of 2000 sq. ft., as illustrated in Fig. 8, the data required for the energy analysis is given in the appropriate areas. This information will include a detailed overview of the structure. The input data needed for the energy analysis should be carefully collected from previous studies^70,71^. The baseline values of the parameters for the building are provided in Table 3.

**Table 3:** Baseline values for a building.

No	Block	Description	Building baseline values
1	HVAC	EER (Energy Efficiency Ratio) as per Breau Energy Efficiency Standards (4-star Rating)	4.24
**2**		The overall efficiency of the supply fan and motor	43%
3		Economizer	None
4	Building envelope	Conventional Reinforced Cement Concrete Roof (150 mm thick with 10 mm thick inside cement plastering)	3.1 W/ m^2^·K
5		Walling System	Based on the wall material as provided in table-3
6		Infiltration	0.3 cfm/sq.ft
7	Windows and doors	U-value	5.0 W/m^2^·K for windows (Single Glazing) 2.5 W/m^2^·K for Wooden doors
8		SHGC (Solar heat gain coefficient)	0.5
9		Overhangs	None
10		Fins	None
11	Lighting	Lighting power density (LPD)	Different for each space
12		Daylight control	None
13	Thermostat setpoint	Cooling setpoint	24 °C
14		Heating setpoint	17 °C
15		Setback control	None
16	Demand controlled ventilation	DCV	None
17	Occupancy	Occupant density	Different for each space
18	Climatic conditions	Dry bulb temperature	As per weather data

The building area was divided into distinct sections, with the living area covering 21%, bedrooms occupying 44%, restrooms taking up 8%, the kitchen accounting for 11%, the dining area covering 12%, and the religious worship area occupying 4%. The wall-to-window ratio was set at 10%. Situated in the warm-humid climate of Visakhapatnam, Andhra Pradesh, India, the building’s location coordinates are Latitude: 17.6868° N and Longitude: 83.2185° E^72^ Simulation studies were conducted based on the U-value of walling materials by considering 200 mm thick wall with 15 mm plastering on both sides has been listed in Table 4. Comparative results were obtained by varying the wall materials, shedding light on the impact of different choices on energy performance. Some of the assumptions are considered for the studies as follows. Firstly, the impact of equipment (chimneys and geysers) layout within the building is assumed to be negligible. Secondly, occupants consistently use the building as per needs and requirements throughout the year. Lastly, it is assumed that equipment operates according to specified schedules of residential buildings in India with vacant timings of 8 am–6 pm throughout the weekdays, 8 am–4 pm on Saturdays, and 4–8 pm on Sundays and holidays^71^.

**Table 4:** Main properties of test material.

Materials	Water absorption (%)	Density (kg/m^3^)	Porosity (%)	28 days Flexural strength (MPa)	28 days Compressive strength (MPa)	*SHC (j/kg k)	*TC (w/m k)	U-Value (W/ m^2^.K)
Clay Bricks	8.1	1819	–	–	25.8	978.6	0.74	2.07
Flyash Bricks	19	1682	–	–	10.14	936	0.52	1.67
TAA Blocks	6.82	2329	8.25	5.07	56.33	1251	0.93	2.81
FTAA Blocks	12.7	1200	15.95	1.98	18.6	831	0.38	1.50

(*SHC = Specific Heat Capacity, TC = Thermal Conductivity, U-Value = Thermal Transmittance Coefficient)

## Results and discussions

### Mechanical performance and XRD analysis

The findings from the experimental program conducted on TAA and FTAA blocks are presented in Table 4, comparing them with commercially available fly ash and clay bricks, commonly used in construction and exhibiting similar performance to the developed blocks^72^. After analyzing the results, it was evident that TAA blocks displayed the highest density and compressive strength among all bricks/blocks, making them suitable for load-bearing structural applications.

However, when considering the utilization of these materials for building facades or insulation, excessive strength becomes unnecessary. To address this, the TAA blocks were subjected to foaming, introducing voids within the material to create FTAA blocks. As a consequence, the compressive strength of FTAA blocks was reduced by approximately half compared to the original TAA blocks, registering at 18.6 MPa. Nevertheless, this strength is still acceptable for structural applications, like commercially used clay and fly ash bricks.

The high compressive strength of TAA blocks can be attributed to the reaction between silica and alumina solids with alkali liquids, resulting in the formation of a geopolymer chain called Silica-Oxygen-Aluminium (Si-O-Al) with alternate sharing of four oxygen atoms (SiO_4_ and AlO_4_)^73^. The gain or increase in strength due to the formation of the polymerization process is due to the formation of calcium-silicate-hydrate (C-S-H) gel and sodium or calcium-alumina-silicate-hydrate (Na or C–A–S–H) gels^74^. The calcium (CaO–34.88%) present in the GGBS also played a significant role in creating a dense microstructure through the formation of additional calcium silicate hydrate or calcium Al-Si hydrate phases in addition to the polymeric chain compounds, which resulted in higher strength performance^14^.

On the other hand, the lower strength observed in FTAA blocks is due to the development of interconnected pores during their preparation employing the foaming agent. The effect of protein-based pre-foaming agent for the development of thermal insulated fly ash-based geopolymer blocks using glass powder. They have achieved a lightweight and also energy-efficient material by increasing the foaming volume by reducing bulk density and thermal conductivity by satisfying the strength requirements as per the Indian standard code provisions^65^. Effect of geopolymer foam on olive oil by using ceramic waste to improve the mix’s physical and mechanical properties^75^. Different percentages of olive oil (0–15 wt%) affect the geopolymer pore formation and composites; as a result, an increase in WA and total porosity is inversely proportional to density and compressive strength decreases due to additive content. Finally, the effect of olive oil results in pore formation and can be used as a foaming agent.

As a result, FTAA blocks exhibit a slightly porous nature and demonstrate lower thermal conductivity, approximately *λ* = 0.38 W/m·K. This reduction in overall thermal properties is a result of facilitating and propagating heat transfer through the pores.

**Fig. 9:**
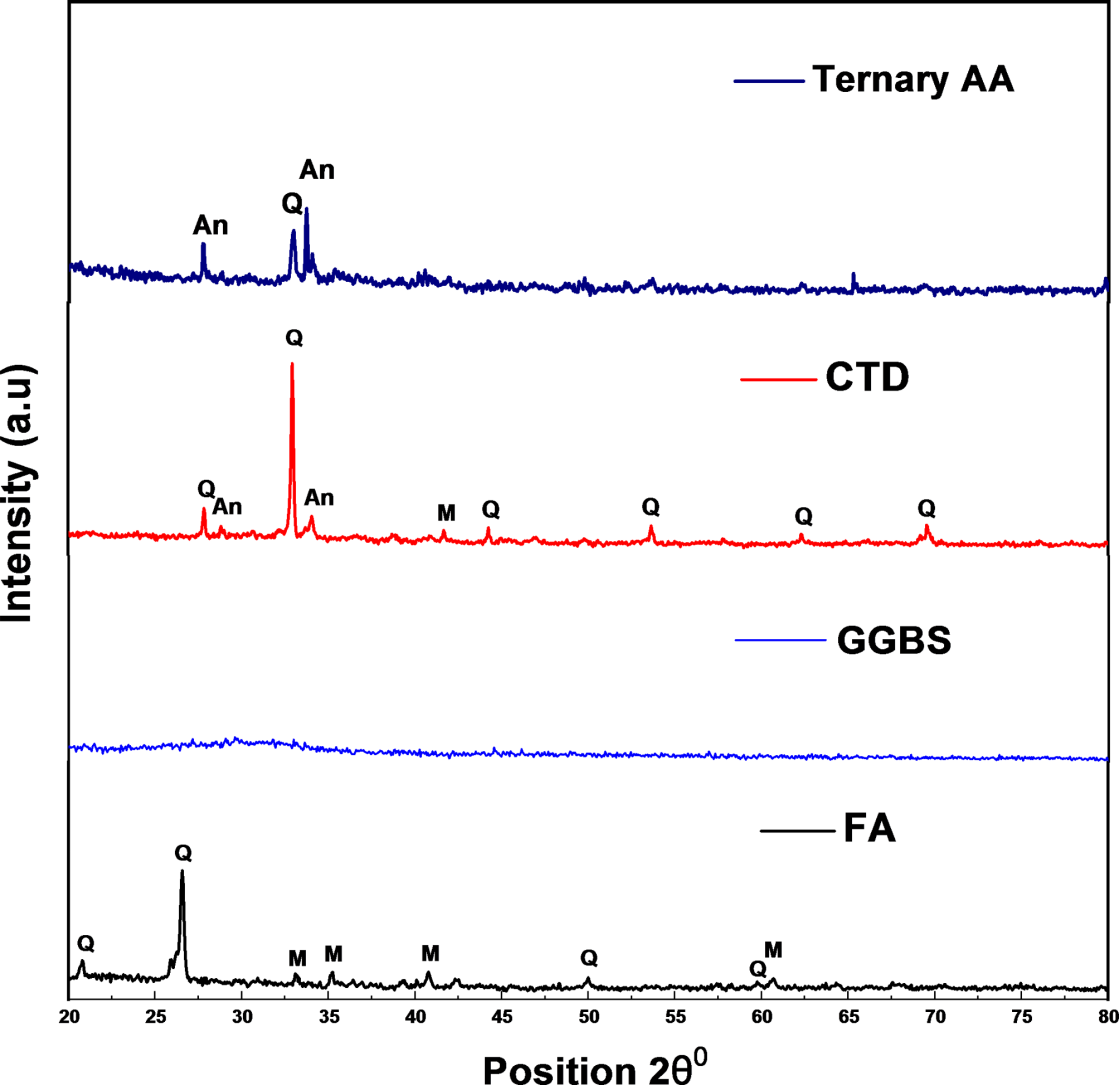
XRD analysis of TAA blocks. (# Q = Quartz, An = Anorthite, M = Mullite).

To investigate the alkali activation process, X-ray diffraction (XRD) analysis was conducted on the raw materials (FA, GGBS, and CTD) and the TAA samples. The XRD analysis utilized Cu Kα1 as the X-ray source, with a step size of 0.0170 and scan angles ranging from 20 to 80° 2θ. By comparing the obtained XRD patterns with existing literature, the minerals present in the samples were identified. This analysis aimed to assess potential changes in the mineral composition of the raw materials during the alkali activation process. Figure 9 displays the XRD for raw FA, GGBS, CTD, and the TAA blocks (referred to as Ternary alkali-activated blocks). The XRD spectra reveal that raw FA and CTD predominantly exhibit crystalline sharp peaks of quartz (Q) and mullite (Mu), alongside a broad hump between 20^0^ 2θ and 30^0^ 2θ. On the other hand, GGBS demonstrates an amorphous nature without any distinct peaks. Notably, XRD spectra depict crystalline peaks of CTD as well as FA on the emergence of new crystalline peaks at approximately 20.8, 26.63, and 27.53 θ^o^ positions.

These new peaks are attributed to the dissolution of the alkali solution, facilitating the formation of new materials^76^. Furthermore, the lower intensity XRD spectra of the TAA material indicate the involvement of raw materials in the alkali activation process, leading to alterations in the original peaks observed in the raw materials^77^. This study highlights the distinctive spectra of each material and demonstrates that the alkali activation process induces changes in their structural characteristics.

### Analysis of building energy performance

The energy demands of indoor spaces are significantly impacted by heat transfer through building components such as walls, windows, and roofs. The building material’s thermal characteristics are crucial in maintaining thermal comfort levels and directly affect the cooling or heating loads of a building. In warm-humid climates like India, coastal regions experience higher temperatures and humidity, necessitating increased cooling for occupant comfort. The National Building Code (NBC, 2005) of India specifies comfort levels based on occupancy, with recommended summer and winter temperatures ranging from 23 to 26 °C and 21 to 23 °C, respectively, at a relative humidity of around 40%. For Visakhapatnam city, the Indian Meteorological Department (IMD) defines the range of temperature-based thermal comfort between 28 and 36.4 °C. This high temperature during summer often leads to reliance on energy-intensive air conditioning systems.

**Fig. 10:**
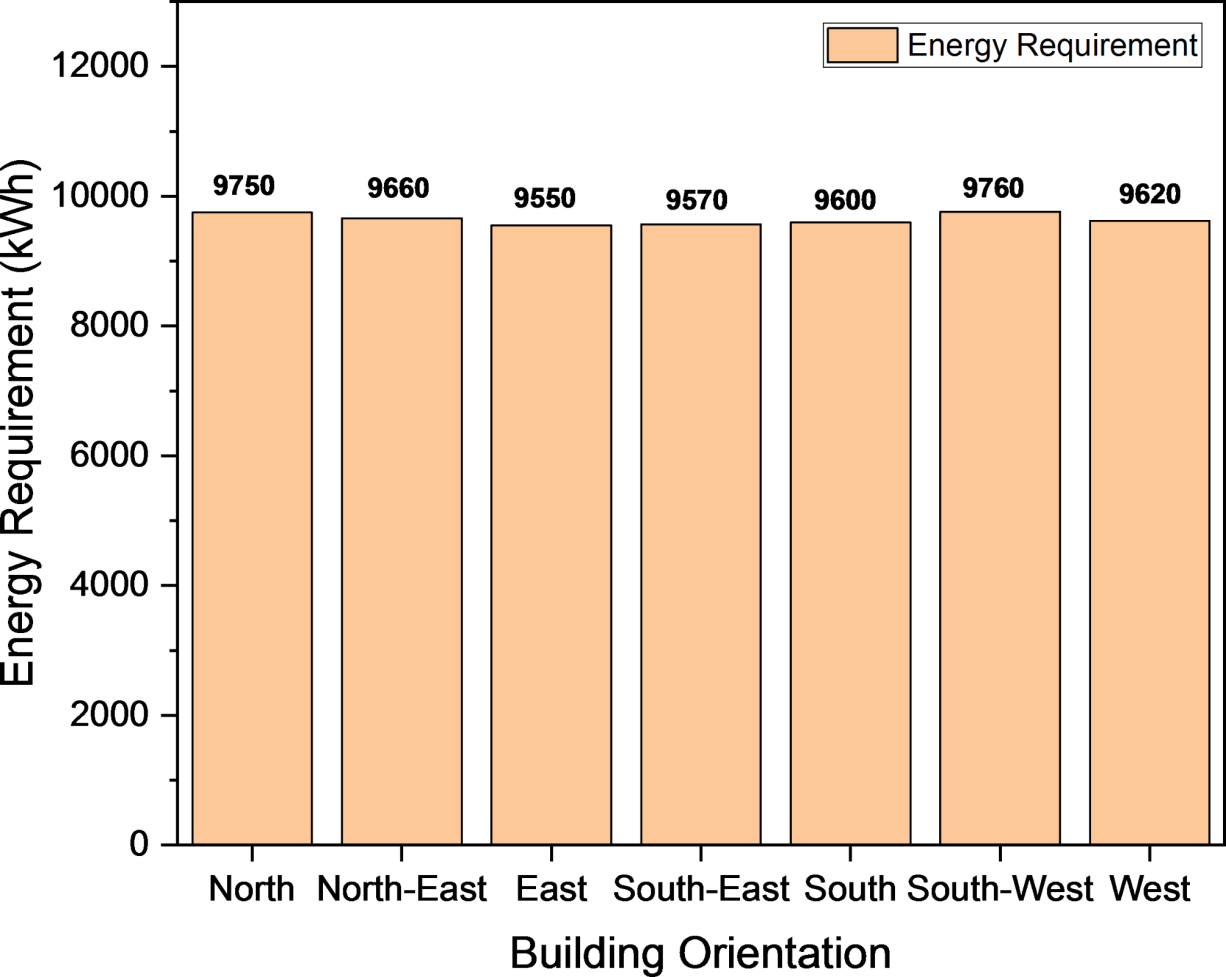
Building Energy Requirement Based on Orientation.

Figure 10 depicts the annual energy requirements of a building based on its orientation, with the east orientation showing slightly lower energy demand (9550 kWh) compared to other orientations. Following the recommended building orientation can directly contribute to energy and cost savings^78^.

**Fig. 11:**
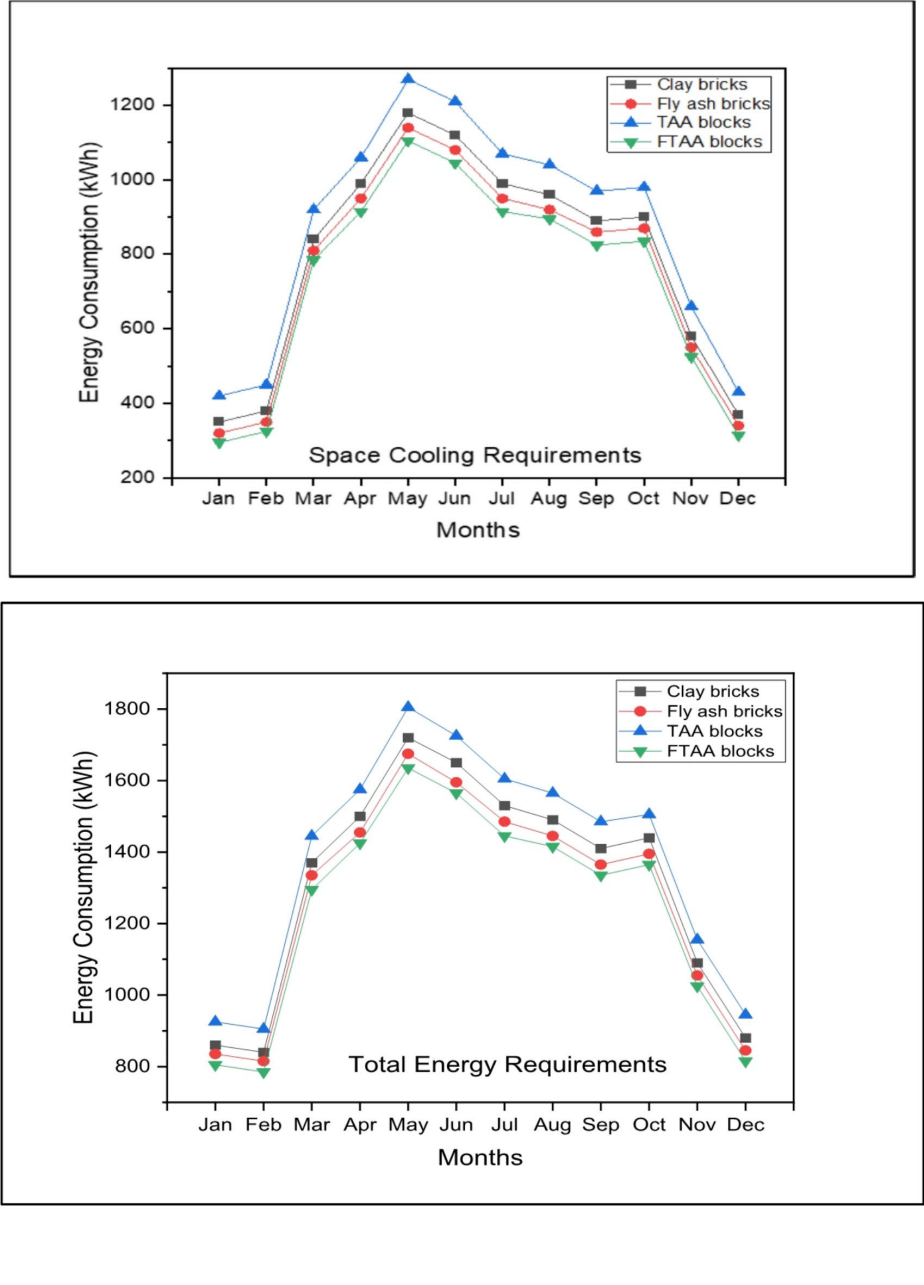
Monthly space cooling energy consumption and TER for different building wall materials.

Figure 11 shows the monthly energy consumption for space cooling, accounting for approximately 55–70% of the TER for various wall systems. The peak monthly energy consumption for space cooling occurs in May, with a demand of 4445 kWh for a building envelope utilizing TAA blocks as wall material. While TAA blocks do not offer significant energy savings compared to conventional red clay bricks, foamed TAA blocks present a potential solution for energy-efficient building envelopes. FTAA blocks exhibit a space cooling energy requirement of 3798 kWh, indicating substantial energy savings compared to TAA blocks.

**Fig. 12:**
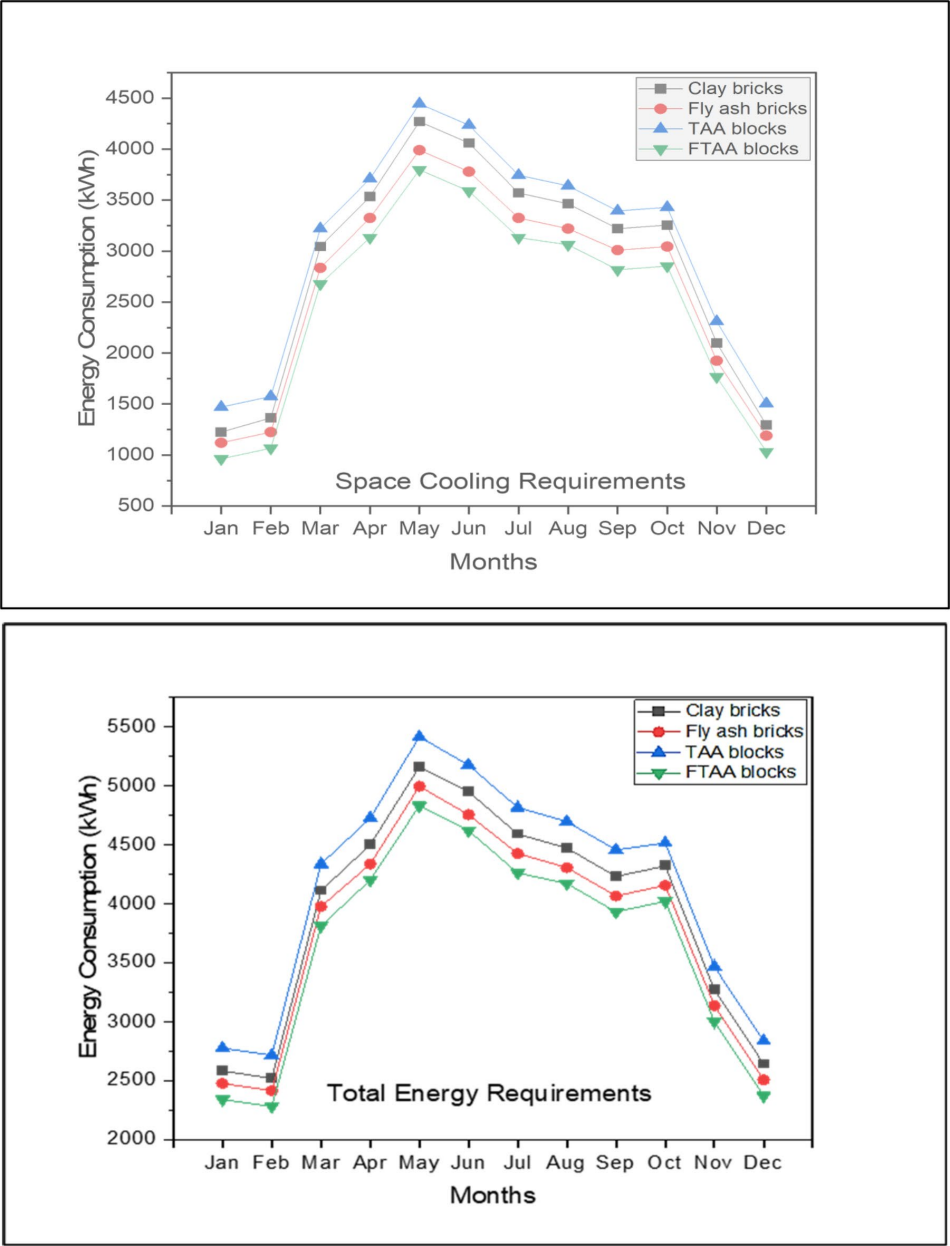
The monthly peak requirement for space cooling and the TER in the case of various building wall materials.

Figure 12 highlights that during the peak month of May, FTAA blocks achieve approximately 11% and 5% total monthly energy savings compared to traditional clay bricks and fly ash bricks, respectively. This underscores the advantage of FTAA blocks in reducing a building’s cooling load dependency due to their minimal heat transmission through the material and wall system.

**Fig. 13:**
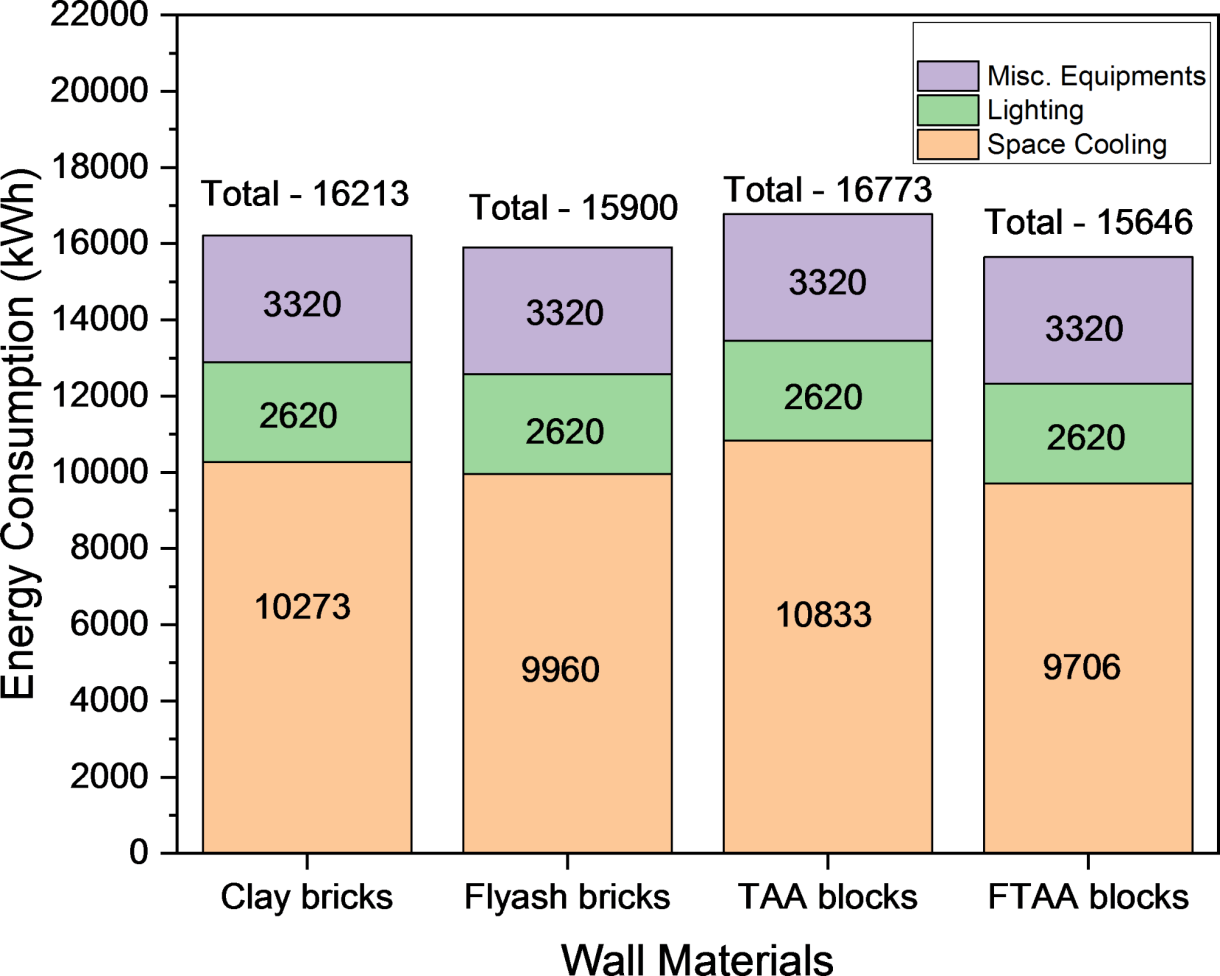
Annual energy consumption of buildings with various wall materials.

**Fig. 14:**
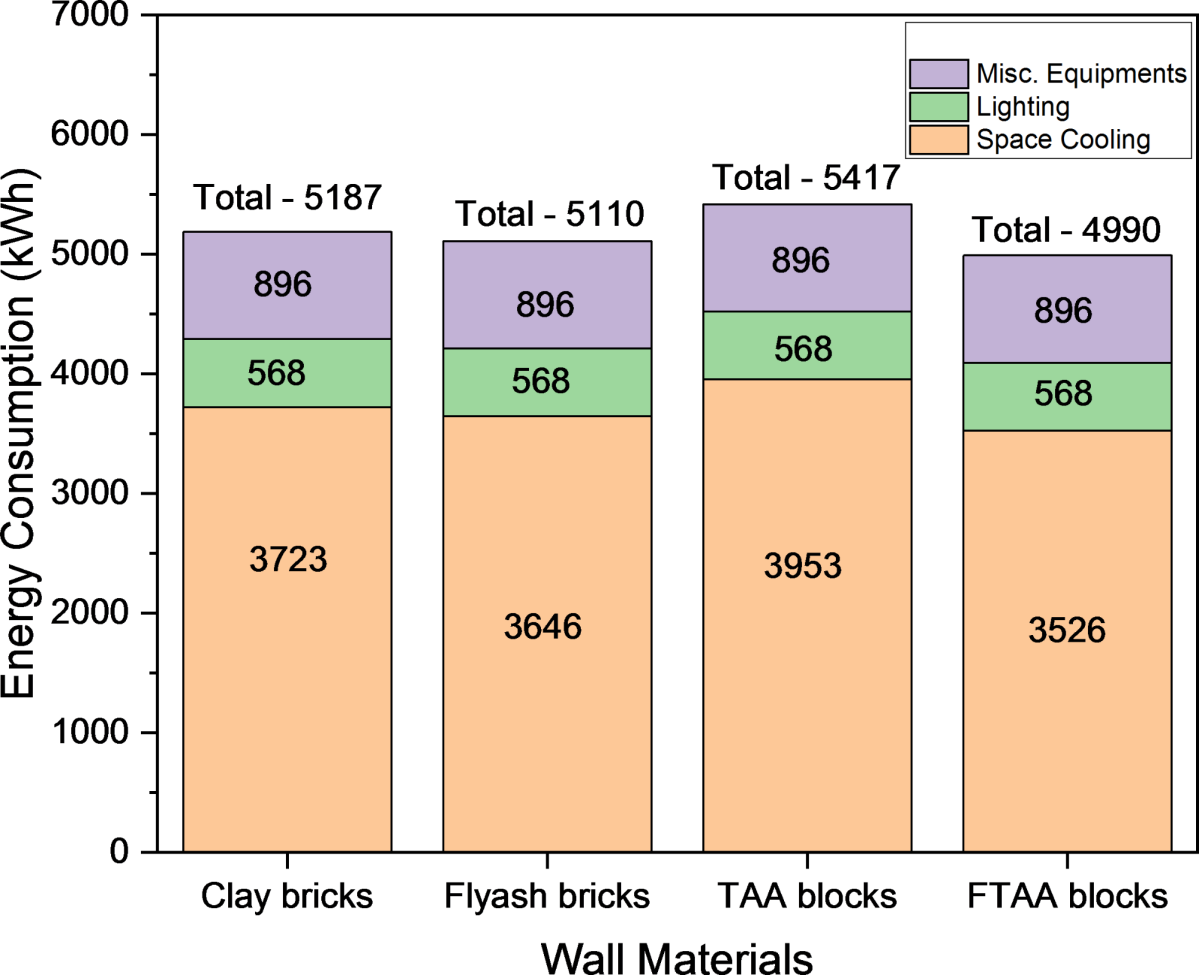
Annual peak energy consumption comparison for buildings with different wall materials.

Figure 13 illustrates the yearly consumption of energy of a building envelope with various wall systems, encompassing air conditioning, lighting, and other miscellaneous equipment. Among the different wall systems, FTAA blocks demonstrate the lowest energy consumption, amounting to 15,646 kWh, signifying their superior thermal performance. The annual energy savings achieved with FTAA blocks are approximately 2% and 4% compared to traditional fly ash and clay bricks, respectively. As per Fig. 14., During peak periods, space cooling contributes to around 58–62% of the overall energy usage in buildings, with occupants heavily relying on air conditioning systems to combat increased temperatures, resulting in higher operational energy demand (i.e., increased electricity consumption). This continuous rise in energy consumption leads to higher carbon emissions and is not sustainable. Thus, adopting FTAA blocks can enhance occupant’s thermal comfort while promoting energy savings, ultimately contributing to the sustainability of the built environment.

**Fig. 15:**
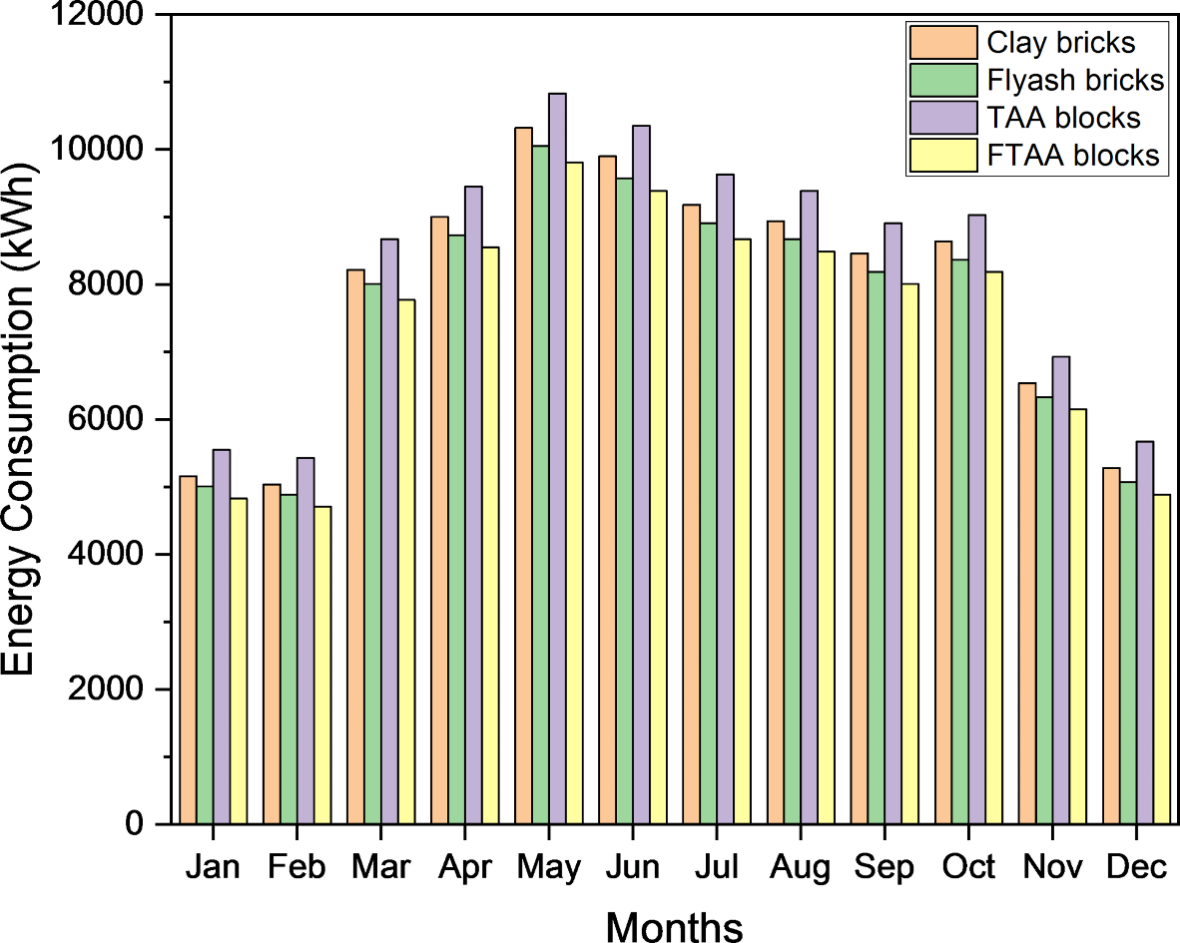
Monthly energy requirement bills for buildings with different wall materials.

The energy consumption and electricity costs of a building are strongly influenced by the equipment usage during its operational stage. Figure 15 presents a comparative analysis of electricity bills based on simulations for buildings with different wall systems. The results clearly demonstrate the significant impact of thermal properties on the building’s energy requirements and operational costs. During the peak month of May, space cooling contributed to higher electricity bills, reaching 9810 kWh for FTAA blocks and 10,320 kWh for conventional clay bricks. The total electricity cost savings during this peak consumption month amount to 4.94%. Moreover, by utilizing FTAA blocks instead of conventional clay bricks, annual electricity bill savings reach 5.51%. Similar trends in energy consumption and cost savings associated with walling and insulation materials have been observed by others^79–81^. These findings underscore the importance of considering energy-efficient materials, such as FTAA blocks, to reduce electricity costs and achieve long-term savings.

**Fig. 16:**
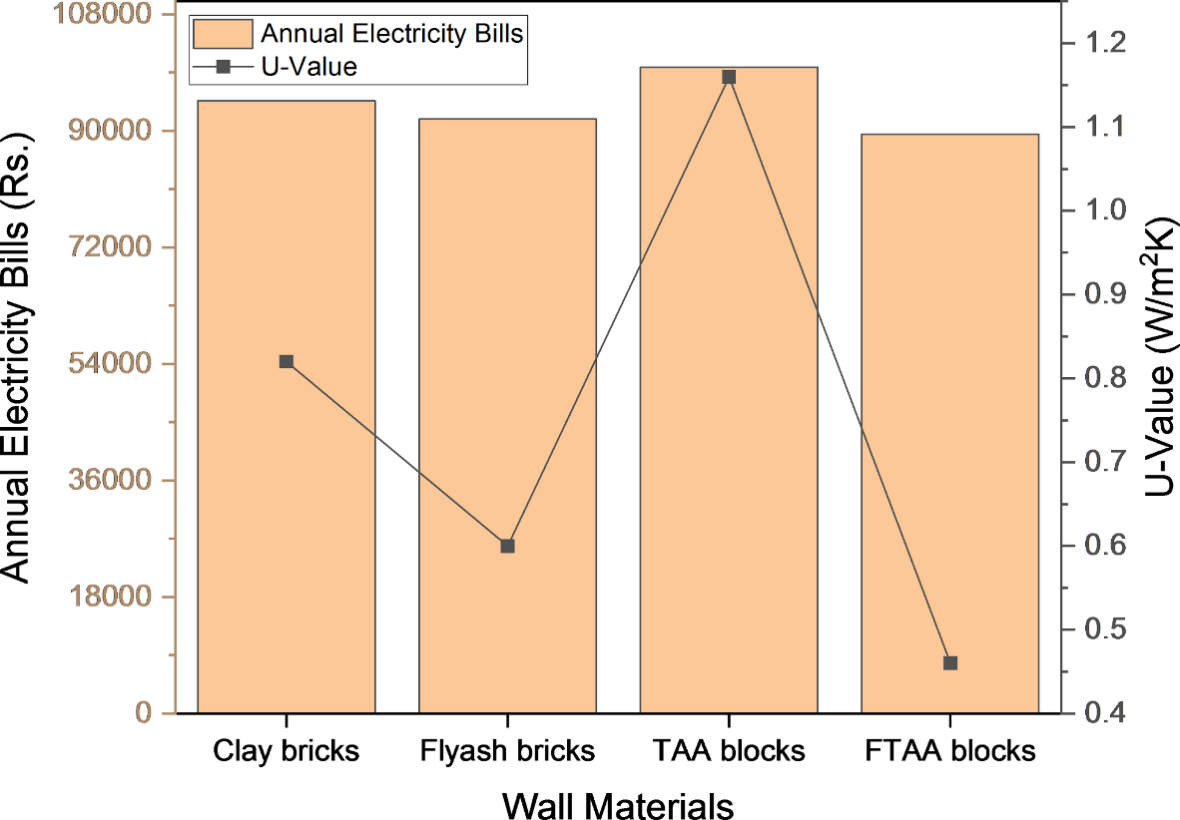
Bills for wall materials.

Figure 16 depicts the correlation between energy costs linked to electricity consumption and the thermal transmittance (U-value) of wall materials. The U-value plays a crucial role in determining the rate of heat transfer through building components. The results indicate that a lower U-value corresponds to a slower rate of heat penetration through the material. Additionally, Fig. 16. provides the U-values of different wall systems composed of various materials. The simulation results highlight that insulated foamed blocks, like FTAA blocks, offer energy savings during the operational stage of a building due to their lower U-value. The insulation capabilities of wall materials, as indicated by their U-values and other thermal properties, can effectively reduce overall electricity consumption and associated costs. Furthermore, minimizing energy usage during building operations significantly contributes to the reduction of carbon footprints^82^.

## Limitations and future scope of the study

### Limitations of the current study

TAA and FTAA block production have many advantages such as it has high strength, low water absorption, low carbon emissions, high thermal stability, and durability. Apart from these advantages, there are a few limitations that need to be addressed to make them useful to a great extent.

For the mass production of these blocks, the following limitations could be observed:

- The availability of raw materials like fly ash, and GGBS may not be possible everywhere to produce TAA or FTAA blocks
- Due to many materials involved in the mix design of TAA or FTAA blocks, there is high sensitivity in mix design. Utmost care should be taken in the control of mix design.
- The perception of the public is still towards clay blocks, usage of these TAA or FTAA blocks needs to be publicized for effective utilization and change of perception of the public.

### Future scope of study

Further research is therefore warranted as mentioned below.

- The performance of other aluminosilicate source materials will be taken for further study.
- AAMs can be studied for various curing ages.
- The performance of alkali-activated materials can be studied further for workability and setting time.
- The durability studies of alkali-activated materials also can be taken up.
- The effect of elevated temperature curing on different alkali-activated materials can be taken up for study.
- The performance of alkali-activated materials can be studied in other curing regimes.
- The feasibility of prefabricated panels can be made for study.

## Conclusions

The research study presents a novel development in the form of ternary alkali-activated blocks and foamed ternary alkali-activated blocks, which offer promising potential as innovative walling materials. The primary emphasis of this research has been on exploring the insulation properties and energy efficiency of the building envelope through the application of FTAA blocks. By comparing these new materials with traditional alternatives, the study has yielded several noteworthy findings and conclusions.

- TAA blocks have higher compressive strength (56.33 MPa) compared to conventional clay bricks (25.8 MPa).
- FTAA blocks demonstrate similar performance to traditional bricks but exhibit significantly lower thermal conductivity (0.38 W/m.K), making them superior as insulation materials.
- Implementation of FTAA blocks can reduce cooling requirements by 11% during the peak month of May compared to clay brick walls.
- Yearly energy savings using FTAA blocks amount to 4%. Cost savings potential includes a 4.94% reduction in expenses during the peak summer month of May and a 5.51% annual cost reduction.
- The development of FTAA blocks signifies a positive step towards adopting greener alternatives to incumbent bricks. This study provides a sustainable solution by reusing waste produced by the ceramic production sector and changing it into an insulated, cost-effective, and ecologically friendly construction material. This is accomplished via the use of research.

## Data Availability

The data that supports the findings of this study are available within the article.
